# Emotional factor matters in language learning? A meta-analysis of emotional intelligence on language achievement

**DOI:** 10.3389/fpsyg.2025.1502112

**Published:** 2025-05-07

**Authors:** Qiao Peng, Li Shuhong

**Affiliations:** ^1^School of Humanities, Hunan City University, Yiyang, China; ^2^Institute of Foreign Languages, Ocean University of China, Qingdao, China

**Keywords:** emotional intelligence, emotional factor, language achievement, second language acquisition, meta-analysis

## Abstract

Emotional intelligence (EI) has garnered sustained theoretical and empirical attention over recent decades. Within the domain of linguistics, a growing body of research has investigated the relationship between EI and language achievement. Publication trends in this area reveal two distinct phases: a period of lukewarm attention (2009–2017), followed by a phase of rapid growth (2018–present). The present meta-analysis aims to determine whether EI significantly influences language achievement. Drawing on data from 47 independent studies, comprising 63 effect sizes and a total sample of 18,649 participants, this study found a small but significant correlation between EI and subjective language achievement (*r* = 0.24), and a moderate correlation with objective language achievement (*r* = 0.41). Moderator analyses revealed that the relationship between EI and objective language achievement varied significantly by educational level, target language, language skill assessed, and publication year. In contrast, no significant moderation effects were found for research type, learning context, students’ major, first language, or the measurement instruments employed. These findings underscore the important role of EI in language learning and highlight the need for emotionally responsive and supportive pedagogical environments that contribute to the sustainable development of foreign language education.

## 1 Introduction

For decades, cognitive abilities have been a central focus in educational research. Cognitive factors such as intelligence quotient (IQ), working memory, processing speed, reasoning, and spatial ability have been widely recognized as key determinants of individual achievement (Sternberg et al., 2001; Rohde and Thompson, 2007; Lu et al., 2011). It was not until the 1970s and 1980s, influenced by the humanistic values of language teaching and learning, that a more holistic perspective on language learners began to emerge—one that emphasized not only cognitive states but also affective factors (Arnold, 1998; Li, 2020). This shift, further propelled by the positive psychology movement (MacIntyre, 2016), brought emotional factors such as emotional intelligence (EI) to the forefront of educational inquiry. A growing body of research suggests that EI may contribute to academic success above and beyond the effects of personality traits and cognitive intelligence (Van der Zee et al., 2002). This perspective has led to what scholars have termed the “emotional turn” in language education, which emphasizes the central role of emotions in the learning process (White, 2018). In this context, EI has been found to play a facilitative role in enhancing various affective and behavioral aspects of language learning, including enjoyment, second language (L2) identity, learning strategies, learning styles, and learner beliefs (Li et al., 2021; Farsad and Modarresi, 2023; Taheri et al., 2019).

However, existing research on the relationship between EI and language achievement has produced inconsistent findings, with reported correlations ranging from positive to negative (e.g., Bagheri and Ghasemi, 2013; Ożańska-Ponikwia et al., 2023). Moreover, the effect sizes documented across studies vary substantially depending on contextual factors. These inconsistencies highlight the necessity of a meta-analytic review to systematically synthesize the evidence and investigate potential moderating variables that may account for the observed variability. Accordingly, the present study aims to conduct a comprehensive and quantitative meta-analysis to examine the overall relationship between EI and language achievement, while also assessing the impact of key moderator variables.

This study distinguishes itself from previous meta-analyses in two key aspects. First, it incorporates a broader range of databases. In response to the expanding body of literature influenced by the positive psychology movement—particularly within the Chinese context (Li, 2020)—this study includes a Chinese-language database. This inclusion addresses the methodological concern that excluding non-English studies may introduce bias into meta-analytic results (Konno et al., 2020). Second, this study adopts more domain-specific criteria for interpreting effect sizes. Rather than relying on Cohen’s (1988) general benchmarks—which classify an *r* value below 0.1 as small, 0.3 as medium, and above 0.5 as large—it follows the recommendations of Plonsky and Oswald (2014), who argue that these thresholds underestimate effect sizes in L2 research. Accordingly, the present study applies interpretation criteria that are more appropriate for the field of applied linguistics. By integrating broader inclusion criteria and employing a context-sensitive analytical threshold, this meta-analysis offers a more nuanced and accurate understanding of the relationship between EI and language achievement. These methodological enhancements contribute to the generation of findings that are both more reliable and more applicable to the domain of language education research.

## 2 Literature review

### 2.1 Emotional intelligence

Emotional intelligence has been defined by various scholars from different perspectives, as summarized in Table 1. Bar-On (1997) conceptualizes EI as a set of abilities that influence a person’s success to cope with environmental demands and pressures. This definition frames EI in terms of an individual’s ability to respond to the environment, emphasizing EI as a form of “ability.” Similarly, Mayer and Salovey (1997) define EI as the ability to monitor the feelings and emotions of oneself and others and to use this information to direct one’s thinking and behavior. They later refined this definition to describe EI as the ability to recognize emotions and their relationships and to reason and solve problems based on emotional information (Mayer et al., 1999). This perspective further underscores EI as a problem-solving “ability.”

**Table 1:** Definitions of EI by different scholars.

Scholar (Year)	Definition
Bar-On (1997)	A set of non-cognitive abilities that influence a person’s success in coping with environmental demands and pressures.
Mayer and Salovey (1997)	The ability to monitor the feelings and emotions of oneself and others and to use this information to direct one’s thinking and behavior.
Mayer et al. (1999)	The ability to recognize emotions and their relationships, and to reason and solve problems based on them.
Petrides (2011)	Trait EI: emotion-related self-perception. Ability EI: emotion-related ability.

Petrides (2011) expands on this by distinguishing between two types of EI: trait EI and ability EI. Trait EI is described as an emotion-related self-perception that is typically measured through self-report instruments, whereas ability EI refers to emotion-related abilities assessed through maximal performance tests. This distinction introduces the “trait” attribute of EI, challenging the purely ability-based interpretations of earlier research. Overall, the existing literature suggests that EI can be conceptualized from two distinct dimensions— “ability” and “trait”—providing a multidimensional framework for understanding its constructs.

Regarding the measurement of EI, numerous psychological scales have been developed, as illustrated in Table 2. One of the most widely used instruments is the *Trait Emotional Intelligence Questionnaire* (TEIQue) developed by Petrides (2009), which includes factors such as emotionality, self-control, sociability, and well-being. This instrument has also been adapted into several forms, including a short form (TEIQue-SF; Cooper and Petrides, 2010), a child form (TEIQue-CF; Mavroveli et al., 2008), and an adolescent form (TEIQue-ASF; Siegling et al., 2015). Another popular measure is the *Emotional Quotient Inventory* (EQ-i), developed by Bar-On (1997), which assesses EI across five dimensions: intrapersonal, interpersonal, stress management, adaptability, and general mood. Subsequent versions include a shorter form (Parker et al., 2011) and a youth version (Bar-On and Parker, 2000). Additionally, *the Self-Report Emotional Intelligence Test* (SREIT), developed by Schutte et al. (1998), assesses EI based on the appraisal and expression of emotions in oneself and others, regulation of emotions, and the utilization of emotions in problem-solving. A more recent development in ability-based EI assessment is the *Mayer–Salovey–Caruso Emotional Intelligence Test* (MSCEIT). Its latest version, *the MSCEIT Research Version* 2.0, evaluates EI across four distinct branches: perceiving emotions, using emotions to facilitate thought, understanding emotions, and managing emotions (Mayer et al., 2003). Taken together, this overview of definitions and measurement tools underscores the evolving conceptualizations of EI and reflects the complexity of the construct, which encompasses both ability-based and trait-based dimensions.

**Table 2:** Different measurements of EI.

Measurement	Factor
TEIQue	Emotionality, self-control, sociability, well-being
EQ-i	Intrapersonal, interpersonal, stress management, adaptability, general mood
SREIT	The appraisal and expression of emotion in self and others, regulation of emotion, utilization of emotion in solving problems
MSCEIT (V2. 0.)	Perceiving emotions, using emotions to facilitate thought, understanding emotions, managing emotions

### 2.2 The control-value theory

The Control-Value Theory offers a foundational framework for understanding the relationship between EI and language achievement within educational settings (Pekrun, 2007). According to the theory, achievement emotions are defined as emotions directly linked to achievement-related activities or their outcomes. These emotions are further classified along two dimensions: valence (positive vs. negative) and object focus (activity-focused vs. outcome-focused). Representative examples include enjoyment, joy, relaxation, anger, boredom, anxiety, and sadness. Achievement, in this context, is defined as outcomes that are evaluated against a standard of excellence. The theory posits that achievement emotions significantly influence achievement: under most conditions, positive emotions are generally associated with positive outcomes, while negative emotions often lead to negative outcomes. This pattern has been observed across multiple academic domains, including language learning and achievement. Although the original version of the theory systematically explains the relationships between these variables, it does not explicitly address the differential roles played by the object focus of emotions.

In the latest update to the Control-Value Theory, Pekrun (2024) emphasizes the importance of object focus, noting that when emotions are directed toward the task itself (e.g., enjoyment), positive activating emotions are more likely to enhance achievement. The updated theory also highlights the nuanced effects of certain emotions. For instance, anxiety, a negative activating emotion, can lead to task-irrelevant thinking and undermine intrinsic motivation, thereby negatively affecting language achievement. However, anxiety can also generate strong extrinsic motivation by increasing effort to avoid failure, which may result in positive effects on language achievement under certain conditions.

Overall, the Control-Value Theory (Pekrun, 2007; Pekrun, 2024) elucidates the close connection between emotions and achievement, while also raising unresolved questions about the role of emotions in educational outcomes—specifically, whether their impact on foreign language achievement is predominantly positive or negative. This ambiguity calls for more systematic and comprehensive research. The theory further prompts new inquiries into how EI influences foreign language achievement, not only regarding the direction of its impact but also its magnitude. Given that EI encompasses the ability or trait to recognize, understand, and regulate emotions, it is plausible to infer that EI could affect language learning outcomes. Investigating this influence is essential for both validating and extending the Control-Value Theory, particularly in the context of foreign language education.

### 2.3 Emotional intelligence and language achievement

In recent years, a growing body of empirical research has examined the relationship between EI and language achievement. One of the earliest studies in this area was conducted by Pishghadam (2009), who surveyed Iranian learners. Since then, the topic has continued to attract scholarly attention (e.g., Ożańska-Ponikwia et al., 2023). Researchers have investigated the EI–language achievement relationship across a variety of countries, educational contexts, academic disciplines, proficiency levels, first languages (L1), and target languages. A wide range of assessment tools has also been employed to measure both EI and language achievement. However, the findings across these studies remain inconsistent, particularly in terms of reported effect sizes. While some studies have identified large effects (e.g., Abdolrezapour, 2018; Abdolrezapour and Tavakoli, 2012; Rokni et al., 2014; Hamdzah et al., 2020; Tabrizi and Esmaeili, 2016), others have reported medium (e.g., Ghaemi and Anari, 2014) or small effect sizes (e.g., Pishghadam, 2009). In addition to differences in magnitude, there are also discrepancies in the direction of the correlation: some studies report a positive association (e.g., Bagheri and Ghasemi, 2013; Manzouri and Movahed, 2017), whereas others suggest a negative relationship (e.g., Rodríguez Prieto, 2010). These divergent findings may be explained, at least in part, by the influence of potential moderator variables that vary across study designs and participant characteristics.

The first potential moderator is *research type*, which explores the consistency between results from published studies (e.g., journal articles) and unpublished studies (e.g., dissertations). This approach aims to identify potential differences between the two, given that published studies may report larger effect sizes (Polanin et al., 2016). The second potential moderator is the *learning context*. Prior research has reported varying effect sizes in second language (SL) and foreign language (FL) learning environments. Learners in SL contexts are generally exposed to more authentic input and are afforded greater opportunities for language use and practice than those in FL contexts, where exposure is often limited to the classroom. For instance, Ding (2022) reported a large effect size in an SL context, whereas Chen and Zhang (2022) observed a small effect size in an FL context. *Educational level* may represent a third moderator variable. Empirical evidence suggests that certain components of EI develop progressively with age, which often correlates positively with educational attainment (Fariselli et al., 2008). As such, learners at higher educational levels may demonstrate more advanced EI, potentially influencing the strength of the EI–language achievement relationship.

The fourth potential moderator is the learners’ *major*. Whether learners study a language major or not determines the amount of time they need to spend on language learning. Different effect sizes were found across different majors. For example, significant large effect sizes were reported in Behjat and Ghasemi’s (2015) study targeting language majors, while small effect sizes and non-significant results were obtained in studies targeting non-language majors (Karimi et al., 2017). The learner’s *first language* (L1) and *target language* could be the fifth and sixth moderating variables. The difficulty of language learning varies due to the differences between L1 and target languages, which can positively or negatively affect language learning (Hsia, 1986). Overcoming these difficulties by managing emotions is necessary for achieving higher levels of achievement, which could lead to varying effect sizes.

The seventh potential moderator is the type of *EI measure*, which is included to examine the reliability and consistency of EI assessments across different measurement instruments. The eighth moderator is the *achievement skill.* Prior research has indicated that the relationship between EI and language achievement tends to yield larger effect sizes for certain skills, particularly speaking and reading (e.g., Ożańska-Ponikwia et al., 2023; Chen and Zhang, 2022). This may be attributed to the fact that higher levels of EI can facilitate more effective management of skill-specific anxieties, such as speaking anxiety and reading anxiety (Ates, 2019). The ninth moderator is the type of *achievement measure* employed. This moderator aims to determine whether various indicators of language achievement—such as course grades, language test scores, academic credits, and GPA—provide consistent and reliable assessments of learners’ achievement. The tenth and final moderator is the *publication year*. This variable is included to explore potential temporal changes in the strength of the relationship between EI and language achievement. The inclusion of this moderator is grounded in the premise that EI, which plays a critical role in stress regulation (Ramesar et al., 2009), may have an increasingly significant impact on managing academic stress over time, thereby influencing language learning outcomes.

## 3 Objectives of the current study

This literature review has examined the definitions and measurement approaches of EI, while also identifying potential moderator variables that may influence the relationship between EI and language achievement. The primary objective of the present study is to investigate the nature and strength of this relationship through a meta-analytic approach. Meta-analysis is a systematic review method that employs statistical techniques to synthesize empirical findings from primary studies within a defined research domain. It is designed to account for sampling errors and the presence of non-significant results, thereby minimizing analytical biases that may stem from subjective interpretation. Owing to its methodological rigor, transparency, and potential for generalizability, meta-analysis has become an increasingly preferred tool for research synthesis—particularly in fields such as education, where evidence-based practices and policies are of growing importance (Vuogan and Li, 2024). Building upon previous research, this study has two specific aims. First, it provides a comprehensive and systematic descriptive analysis to examine publication trends and reporting practices, offering an overview of the current research landscape. Second, it undertakes a detailed meta-analytic investigation to explore situational and linguistic moderator variables that may shape the relationship between EI and language achievement, thereby contributing to a more nuanced and context-sensitive understanding of this association.

In line with these objectives, the study seeks to address the following two research questions:

Research Question 1: What are the publication trends and reporting practices involved in studies of the relationship between EI and language achievement?

Research Question 2: How do *research type, research context, educational level, major, L1, target language, EI measure, achievement skill, achievement measure* and *publication year* moderate the relationship between EI and language achievement?

## 4 Methods

### 4.1 Literature search

To minimize potential publication bias and ensure a more representative and comprehensive sample, this meta-analysis includes both published and unpublished studies (Norris and Ortega, 2006). The inclusion of unpublished research is consistent with the recommendation by Polanin et al. (2016), who notes that published studies tend to report inflated effect sizes due to publication bias. By incorporating unpublished studies, this meta-analysis aims to mitigate such bias and yield a more balanced and accurate estimation of the true effect size.

Following the guidelines proposed by Plonsky and Brown (2015), several methods were employed to identify literature examining the relationship between EI and language achievement. First, a comprehensive search was conducted across multiple online databases, including the Education Resources Information Center (ERIC), Linguistics and Language Behavior Abstracts (LLBA), Web of Science (WOS), ProQuest, Google Scholar, and China National Knowledge Infrastructure (CNKI). The search string used was “(emotion* intelligence OR emotional intelligence OR emotional quotient) AND (achievement OR proficiency OR attainment OR performance) AND (L2 OR foreign language OR second language OR language OR EFL OR SLA).” Second, a manual search strategy was adopted to ensure comprehensive coverage of relevant literature (Perera and DiGiacomo, 2013). This included targeted searches of key journals such as *Frontiers in Psychology, System, Modern Language Journal, Language Learning, Language Teaching Research,* and *Studies in Second Language Acquisition*. No restrictions were imposed on publication year, and both English and Chinese language publications were considered. Finally, backward and forward citation searches were conducted based on seminal and recent articles. This search process yielded a total of 1,653 citations.

### 4.2 Exclusion criteria

To determine whether the identified literature aligns with the research question, the title, abstract, and full text of each study were carefully reviewed. Five exclusion criteria were applied to filter the studies: (1) the study must be published in English or Chinese. (2) The study must report at least one measurement tool for both EI and language achievement. EI measures must be based on self-report scales, and language achievement must pertain to SL or FL achievement. Studies focused on non-language subjects (e.g., Mathematics) or those that did not provide comprehensive achievement scores (e.g., reporting only complexity and fluency of spoken English without a total spoken English score) were excluded. (3) The relationship between EI and language achievement must be reported in the form of a correlation or another statistic (e.g., t or *F*) that can be converted into effect sizes (e.g., correlation coefficients). Studies employing linear regression or structural equation modeling were excluded unless they reported correlation statistics. (4) The study participants must be recruited from educational settings (e.g., studies in hospital contexts were excluded), and the participants must be SL/FL learners. (5) To maintain consistency, only cross-sectional studies were included, while longitudinal studies were excluded.

**Figure 1:**
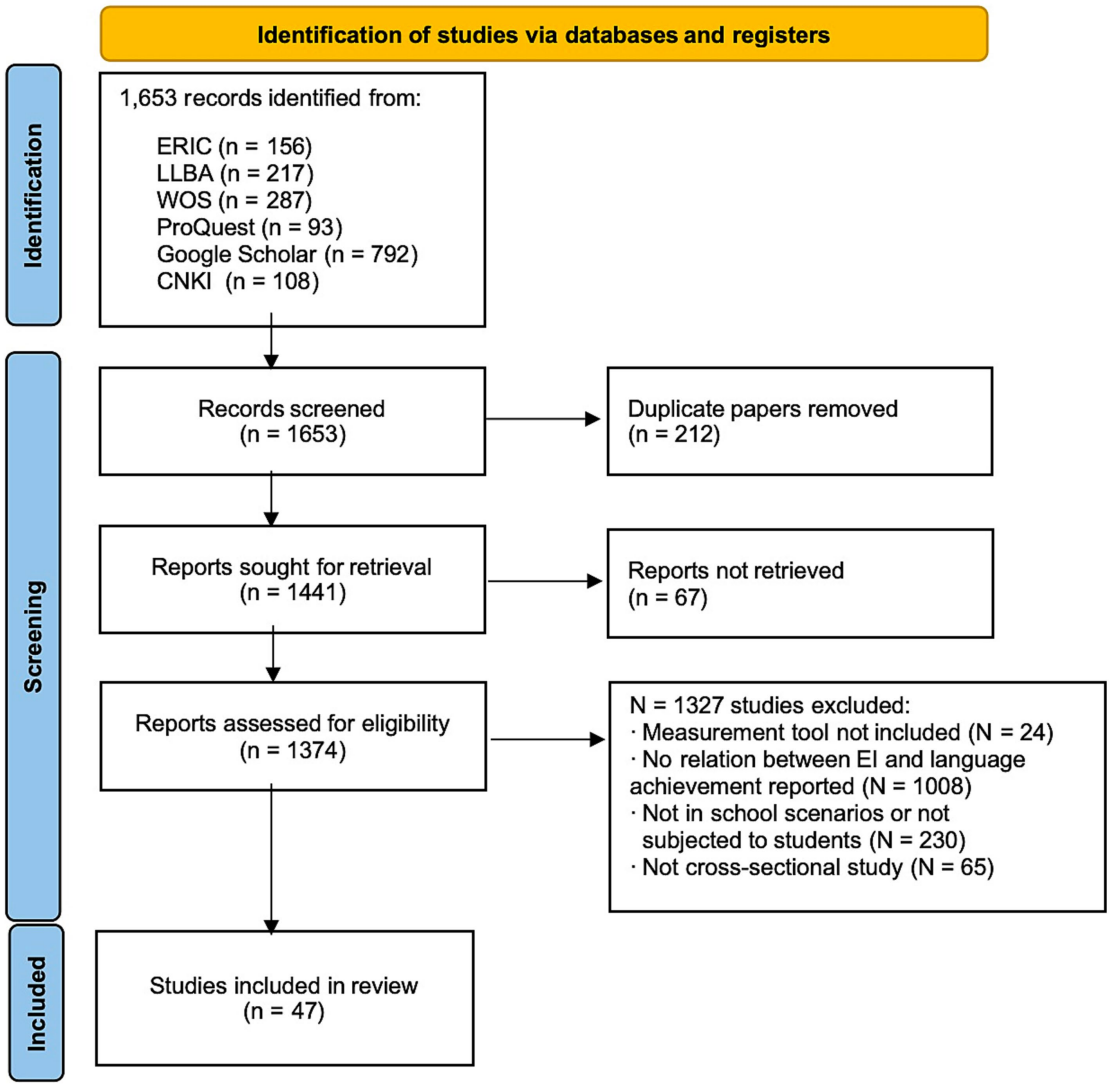
Database search and selection flowchart.

After applying these criteria to the initial pool of literature, a total of 47 studies (33 journal articles and 14 dissertations) were retained for further analysis. The flowchart outlining the database search and selection process is presented in Figure 1.

### 4.3 Coding procedures

To systematically capture the detailed characteristics of the selected studies, a comprehensive coding scheme was developed, as presented in Table 3. This scheme comprises six major categories for analysis: (1) bibliographic information, (2) research design, (3) participant, (4) instrumentation (EI), (5) instrumentation (language achievement), and (6) reported results.

**Table 3:** Variables and definitions of items in the coding scheme.

Variable	Definition
Bibliographic Information	
Author(s)	Author(s) who conduct(s) and publish the research
Title	Title of the study
Year	Year in which the study was published
Type	Type of research (journal; dissertations; book chapters; conference proceedings; unpublished manuscripts)
Research Design	
Context	Language context in which the research was conducted (SL; FL; study abroad)
Country	Country in which the study was carried out
Participant	
Sample size	Sample size of participants in the study
Mean age	Mean age of participants participating in the study
Female ratio	Percentage of all female participants in the study
Educational Level	Educational level at which the participant is engaged (primary school; middle school; high school; undergraduate; postgraduate; institute)
Proficiency level	The language proficiency level of the participants (beginning; intermediate; advanced; multiple)
Major	Whether the subject studied in a language major (language major = LM; non-language major = non-LM)
L1	First language of the subjects
Target language	Target language of subjects
Instrumentation (EI)	
Type	The type of EI (ability EI; trait EI)
Scale	Scales used to measure EI
Instrumentation (Language Achievement)	
Skill	Skills related to measured language achievement (general skill; listening; speaking; reading; writing; vocabulary; mixed skill)
Type	Types of language achievement (subjective achievement; objective achievement)
Measure	The type of language achievement measure used in the study (course grade; language test; GPA; credit; self-perceived competence)
Result	
Reported effect size (*r*)	Observed correlation between EI and achievement

In line with the methodology proposed by Plonsky and Oswald (2014), independent effect sizes were coded when studies reported separate results for distinct sub-samples (e.g., males and females, middle school and high school students). The coding scheme underwent several rounds of refinement before its implementation to ensure clarity and consistency. Initial inter-coder reliability was calculated at 97%. After resolving discrepancies through detailed discussions between the two coders, the final inter-coder reliability reached 100%.

### 4.4 Data analysis

Statistical Product and Service Solutions (SPSS) v27.0 and Comprehensive Meta-Analysis (CMA) v2.0 (Hunter and Schmidt, 2004) were employed for data analysis. To address Research Question 1, the study began with a detailed descriptive analysis of publication trends and reporting practices, which encompassed study design, context, country, sample size, mean age, female ratio, educational level, proficiency level, major, L1, target language, and instrumentation.

To answer Research Question 2, a random-effects model was utilized to account for the variability in study characteristics across studies. Weighted means of all effect sizes, along with their 95% confidence intervals (CIs), were computed. Additionally, potential publication bias was assessed. The heterogeneity analysis included calculating the goodness-of-fit statistic (*Q*) and evaluating systematic variation between observed effects (*I*^2^), in line with the assumptions of moderated effects (Cooper et al., 2019). Potential moderator variables were analyzed through subgroup analyses and meta-regression.

## 5 Results

### 5.1 Descriptive analysis of the publication trend and reporting practices

**Figure 2:**
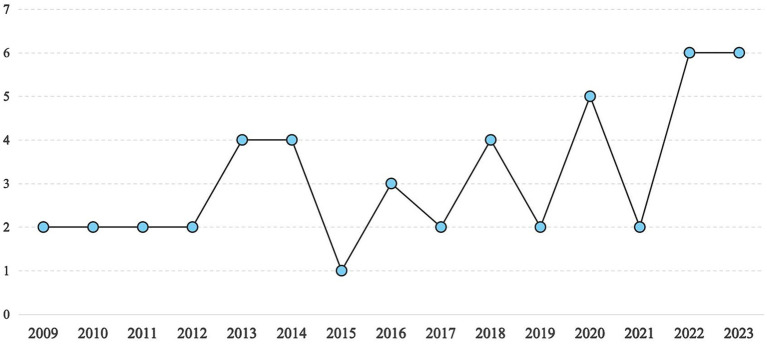
The description of literature published in each year.

The 47 studies included in this research sample were conducted across eight countries between 2009 and 2023. The sample comprised a total of 63 effect sizes (*r*) and included 18,649 individual participants. The first research question focuses on the publication trends and reporting practices within the sample literature. Figure 2 illustrates the annual trends in publication. The distribution of articles was as follows: 1 article in 2015 (2.13%), 2 articles each in 2009, 2010, 2011, 2012, 2017, 2019, and 2021 (4.26% each), 3 articles in 2016 (6.38%), 4 articles each in 2013, 2014, and 2018 (8.51% each), 5 articles in 2020 (10.64%), and 6 articles each in 2022 and 2023 (12.77% each). As of now, no studies meeting the selection criteria have been published in 2024. In this meta-analysis, each independently coded sample served as the unit of analysis rather than the study itself. Given that some studies reported multiple independent samples with distinct characteristics, descriptive statistics were computed based on the 63 independent effect sizes. The following section summarizes the sample characteristics. Regarding publication type, the majority of the literature consisted of journal articles (*N* = 49, 77.7%), while a smaller proportion were dissertations (*N* = 14, 22.2%).

All studies included in this research sample utilized a cross-sectional design. The majority of independent samples focused on FL contexts (*N* = 45, 71.4%), followed by SL contexts (*N* = 14, 22.2%) and mixed contexts (*N* = 3, 4.7%). One study (1.5%) did not specify the context. The research covered language learners from several countries, including Iran (*N* = 26), China (*N* = 25), Malaysia (*N* = 1), Poland (*N* = 5), Saudi Arabia (*N* = 1), Tunisia (*N* = 1), the United States (*N* = 2), and multi-country learners (e.g., United Kingdom and United States). One study (1.5%) did not report the nationality of the subjects.

Regarding participant characteristics, sample sizes ranged from 30 to 1,718 (M = 296, SD = 365.55). Twenty-one independent samples (33.33%) reported mean ages ranging from 14 to 39, while 42 independent samples (66.66%) did not provide mean age data. Fifty-one independent samples (80.95%) reported the proportion of female participants, with female ratios ranging from 33.33 to 100%. Twelve independent samples (19%) did not provide data on the number of female participants. Additionally, 62 independent samples (98.41%) described the educational levels of learners, which included middle school (*N* = 4, 6.3%), high school (*N* = 12, 19%), undergraduate (*N* = 36, 57.1%), postgraduate (*N* = 5, 7.9%), institute (*N* = 4, 6.3%), and mixed education levels (e.g., high school and undergraduate, *N* = 1, 1.5%). One study (1.5%) did not report the educational level.

Furthermore, only 18 independent samples (28.57%) reported participants’ proficiency levels, categorized as beginning (*N* = 1, 1.5%), intermediate (*N* = 6, 9.5%), advanced (*N* = 1, 1.5%), and multiple levels (*N* = 10, 15.8%). Forty-five independent samples (71.4%) did not report proficiency levels. Fifty independent samples (79.36%) specified participants’ majors, including language major (*N* = 14, 22.2%), non-language major (*N* = 33, 52.3%), and mixed majors (*N* = 3, 4.7%). Thirteen independent samples (20.6%) did not report participants’ majors. Forty-four independent samples (69.84%) reported participants’ first languages (L1), including Chinese (*N* = 25, 39.6%), Iranian (*N* = 5, 7.9%), Persian (*N* = 8, 12.6%), Turkish (*N* = 1, 1.5%), and mixed languages (*N* = 5, 7.9%). Nineteen independent samples (30.1%) did not report L1. Participant’ target languages were reported in all studies, including English (*N* = 61, 96.8%) and Spanish (*N* = 2, 3.1%).

In terms of instrumentation, the literature predominantly measured trait EI rather than ability EI, as only trait EI can be assessed via self-report (Petrides, 2009). The scales used to measure EI varied, including TEIQue (*N* = 31, 49.2%), EQ-i (*N* = 22, 34.9%), and SREIT (*N* = 10, 15.87%). Language achievement was assessed in various forms, including general skills (*N* = 32, 50.7%), listening (*N* = 5, 7.9%), speaking (*N* = 8, 12.6%), reading (*N* = 7, 11.1%), writing (*N* = 5, 7.9%), vocabulary (*N* = 5, 7.9%), and mixed skills (e.g., listening and reading, *N* = 1, 1.5%). Language achievement was classified as subjective (*N* = 9, 14.2%) or objective (*N* = 54, 85.7%). It was primarily measured by language tests (*N* = 35, 55.5%), followed by course grades (*N* = 17, 26.9%), self-perceived competence (*N* = 9, 14.2%), credit (*N* = 1, 1.5%), and GPA (*N* = 1, 1.5%).

### 5.2 Meta-analysis of potential moderator variables

Research Question 2 examines the moderating variables affecting the relationship between EI and language achievement. To ensure data independence, the data were divided into two sub-datasets based on the type of language achievement measure: subjective and objective.

Among the nine studies assessing subjective achievement, eight reported a positive correlation, while one reported a negative correlation. The inverse-variance weighted mean for subjective achievement indicated a small effect size (weighted *r* = 0.24, 95% CI: [0.24–0.35]; Plonsky and Oswald, 2014), suggesting a positive yet small relationship between EI and subjective language achievement. In contrast, of the 54 studies on objective achievement, 52 reported a positive correlation and two reported a negative correlation. The inverse-variance weighted mean for objective achievement revealed a significant positive correlation with a moderate effect size (weighted *r* = 0.41, 95% CI: [0.34–0.47]), indicating a positive and moderate relationship between EI and objective language achievement. A detailed overview of these effects is provided in Appendix A. Given the predominance of studies measuring objective achievement, subsequent analyses will focus on the 54 independent samples that assessed objective achievement.

**Figure 3:**
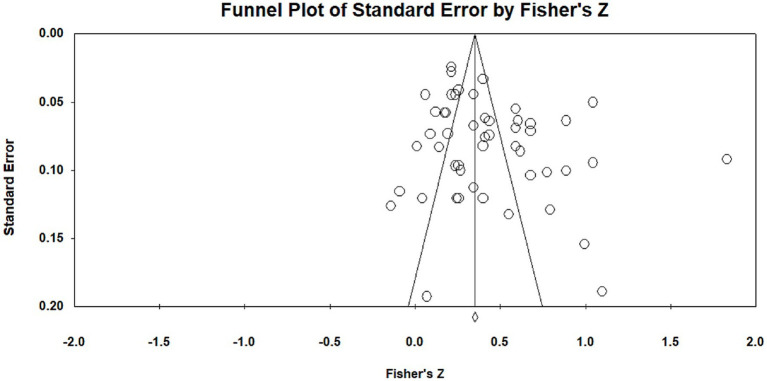
Funnel plot of effect sizes.

To investigate potential publication bias, a funnel plot was constructed to illustrate the relationship between effect size and standard error. A symmetrical, funnel-shaped distribution typically indicates no significant publication bias (Borenstein et al., 2021). As shown in Figure 3, most effect sizes are clustered around the mean; however, several data points are positioned to the right of the plot, suggesting the possibility of slight publication bias. To further assess this issue, a classic fail-safe *N* analysis was conducted to estimate the number of additional studies with null results that would be needed to overturn the findings of this meta-analysis (Cooper et al., 2019). The analysis produced a fail-safe *N* of 5,188, indicating that it would take 5,188 unpublished or missing studies with null effects to meaningfully alter the current conclusions. Given the improbability of such a large number of omitted studies, it is reasonable to conclude that there is no obvious or pronounced publication bias in this meta-analysis. In addition, Kendall’s rank correlation test yielded a two-tailed *p*-value greater than 0.05, providing further evidence against the presence of significant publication bias and reinforcing the conclusion that no clear or systematic publication bias is apparent in this study.

The heterogeneity test revealed a significant within-study goodness-of-fit statistic (*Q* = 1148.688, *p* < 0.05), indicating substantial heterogeneity among the effect sizes. Furthermore, 95.39% of the observed variation between effect sizes was attributable to factors other than sampling error (*I*^2^ = 95.39). This suggests that the observed heterogeneity may be attributed to the influence of moderating variables, thereby warranting further moderator analyses. To identify these moderator variables, subgroup analyses and meta-regression were conducted across 10 categories, as detailed in Table 4. The results indicated that variables such as *research type, research context, major, L1, EI measure*, and *achievement measure* did not significantly affect the effect size of the relationship between EI and language achievement (*p* > 0.05). Conversely, *educational level, target language, achievement skill*, and *publication year* were found to be significant moderators influencing effect sizes (*p* < 0.05).

**Table 4:** The results of moderator analyses.

Categorical moderators			95% CI		
	*k*	*r*	Lower	Upper	*Q*_between_
Research Type					0.16
Journal	41	0.41	0.33	0.49	
Dissertation	13	0.39	0.26	0.49	
Research Context					4.29
SL	14	0.40	0.28	0.51	
FL	36	0.39	0.30	0.47	
Mixed	3	0.64	0.12	0.88	
Missing	1	0.23	0.05	0.4	
Educational Level					63.04^***^
Middle School	4	0.40	0.26	0.51	
High School	10	0.51	0.33	0.66	
Undergraduate	29	0.38	0.28	0.46	
Postgraduate	5	0.19	0.06	0.32	
Institute	4	0.65	0.56	0.72	
Mixed	1	0.14	−0.02	0.29	
Missing	1	0.18	0.07	0.29	
Major					2.32
LM	14	0.33	0.23	0.42	
Non-LM	24	0.40	0.31	0.48	
Mixed	3	0.62	0.06	0.88	
Missing	13	0.43	0.18	0.64	
L1					2.39
Chinese	22	0.34	0.26	0.42	
Persian	13	0.43	0.29	0.55	
Others	6	0.53	0.02	0.82	
Missing	13	0.44	0.27	0.58	
Target Language					35.57^***^
English	52	0.42	0.35	0.49	
Spanish	2	−0.11	−0.27	0.05	
EI Measure					6.39
TEIQue	22	0.32	0.26	0.38	
EQ-i	22	0.51	0.38	0.62	
SREIT	10	0.35	0.13	0.53	
Achievement Skill					15.27^*^
General	27	0.37	0.29	0.45	
Listening	4	0.37	0.17	0.55	
Speaking	7	0.49	0.31	0.63	
Reading	6	0.54	0.08	0.81	
Writing	4	0.30	−0.21	0.68	
Vocabulary	5	0.33	0.09	0.54	
Receptive Skills	1	0.80	0.62	0.90	
Achievement Measure					3.99
Course Grade	17	0.37	0.26	0.47	
Language Test	35	0.43	0.33	0.51	
Credit	1	0.23	0.05	0.40	
GPA	1	0.38	0.24	0.51	

Continuous moderators			95% CI		
	*β*	SE	Lower	Upper	*Q*_between_
Publication Year	0.007	0.001	0.004	0.01	18.67^***^

^*^p < 0.05. ^***^p < 0.001.

Regarding *educational level*, the largest effect sizes were observed in studies focusing on institute-level learners, followed by high school, middle school, undergraduate, and postgraduate levels. Due to the limited number of studies covering middle school, postgraduate, and institute levels, these results should be interpreted with caution. For *L1*, the effect size remained consistent across studies with Chinese, Persian, and other languages. Concerning the *target language*, effect sizes were larger for studies where English was the target language compared to those with Spanish. Given the limited literature on Spanish as the target language, caution is advised in interpreting these findings. In terms of language *achievement skills*, the highest effect sizes were found in receptive skills (i.e., listening and reading), followed by reading, speaking, listening, general skills, vocabulary, and writing. Given the limited literature on specific skills, except for general skills, these results should be approached with caution. Lastly, effect sizes tended to be larger for more recently published studies.

## 6 Discussion

Over the past two decades, a substantial body of research has explored the relationship between EI and language achievement. This study aims to provide a systematic meta-analysis, offering both a broad overview and a detailed examination of this body of work.

### 6.1 Publication trends and reporting practices

The first research question aimed to examine overall trends and reporting practices in the literature on this topic. An analysis of annual publication patterns reveals a gradual but uneven increase in scholarly attention, which can be divided into two distinct phases: a period of lukewarm attention (2009–2017) and a phase of rapid growth (2018–present). The initial phase was marked by relatively modest output, while the surge in publications since 2018 may be attributed to the emergence of the “emotional turn” in applied linguistics (White, 2018) and the rise of positive psychology (MacIntyre, 2016), both of which have shifted research focus beyond purely cognitive factors.

Given that the majority of the reviewed studies targeted English as the L2, this trend may be attributed to the fact that English is commonly taught as a second or foreign language in many countries’ educational systems. Moreover, the analysis of reporting practices reveals that research contexts and countries were generally well-documented, with the majority of studies conducted in Iran and China. This geographical concentration may be explained by two primary factors. First, the inclusion of both English-language international journals and Chinese-language publications helped mitigate potential “English bias” (Konno et al., 2020), thereby increasing the representation of studies from China. Second, cultural emphasis on academic achievement in both countries likely contributes to the research interest in this area. In China, Confucian educational philosophy emphasizes the role of norms and standardized examinations as key indicators of academic competence (Yan, 2019). Similarly, Iran’s educational culture, shaped by Islamic traditions, places a high value on knowledge acquisition and relies heavily on summative assessments to evaluate academic performance (Zarrinabadi and Mahmoudi-Gahroei, 2018).

While the existing literature has predominantly focused on learners in Chinese and Iranian contexts and on English as the target language, this does not undermine the generalizability or applicability of the present findings. Many educational settings worldwide share comparable characteristics—learners often face challenges in acquiring English as a foreign or second language, and academic achievement remains a central concern in both institutional policies and societal expectations. In this regard, the study offers valuable insights that may extend to other contexts, provided similar research efforts are undertaken. EI, as a universal human trait or state, transcends linguistic and cultural boundaries (Petrides, 2011). Therefore, findings from research conducted primarily in China and Iran can still inform teaching practices in other regions, offering implications for the integration of emotional and cognitive dimensions in language education globally.

A portion of existing studies does not report some participant details, such as mean age, proficiency level, and L1, which mildly limits the generalizability of findings and hinders the broader application of meta-analytic results. Since the difficulty of language learning varies depending on the linguistic distance between L1 and the target language (Hsia, 1986), and EI manifests differently across developmental stages and language proficiency, reporting these factors is essential for capturing the full complexity of the learning process. Enhanced reporting would not only improve transparency and replicability but also enable more accurate evaluations of potential moderating effects. This study underscores these reporting gaps, providing a foundation for future research in this area. There is a noticeable disparity in the reporting of EI and language achievement measures. The TEIQue and EQ-i are more commonly used than the SREIT, likely due to the availability of multiple validated versions of the former two. Furthermore, studies predominantly focus on general language achievement, with limited attention to specific skills such as listening, reading, and writing, often due to the scarcity of well-established assessment tools for these areas. There is also a stronger emphasis on objective measures, such as language tests, over subjective evaluations, possibly due to their perceived validity and authority.

### 6.2 Moderating variables

The second research question focused on identifying moderator variables that influence the relationship between EI and language achievement. The findings revealed that EI has a small effect on subjective achievement and a moderate effect on objective achievement. This suggests that higher levels of EI are associated with more positive self-perceptions of achievement as well as greater actual success in language learning. These results are consistent with the control-value theory (Pekrun, 2007; Pekrun, 2024), which highlights the influence of emotional factors on academic outcomes. Moreover, comparisons between different types of language achievement measures showed that the effect sizes were larger for objective achievement than for subjective achievement. This disparity may be explained by the role of EI in enhancing learners’ ability to regulate emotions and manage stress, which directly impacts performance in assessment contexts. Specifically, learners with higher EI are better equipped to employ effective test-taking strategies, reduce test anxiety, and maintain focus, thereby achieving higher scores in objective evaluations. These findings support the view that emotionally related factors are critical to learning success (Fredrickson, 2001), and underscore the stronger influence of EI on objective language achievement.

The analysis indicated no significant differences in the EI-language achievement relationship between journal articles and dissertations, suggesting consistency across published and unpublished studies in this research. Additionally, the impact of EI was consistent across SL and FL contexts, demonstrating that EI maintains its significance regardless of the learning context. However, moderating effects were observed based on *educational level*, with the strongest relationships found at educational stages not focused on research development (e.g., middle school, high school, undergraduate, and institute). The relationship was weaker at the postgraduate level, which prioritizes research skills. This finding suggests that learners with a stronger research orientation may adopt a more analytical approach to learning, potentially diminishing the impact of EI. This result challenges the notion that EI consistently increases with educational level (Fariselli et al., 2008) and underscores the importance of considering how a research-oriented mindset influences learning.

The relationship between EI and language achievement did not exhibit significant variation based on whether students were majoring in language studies. This suggests that EI remains a crucial factor in language learning regardless of the student’s field of study or the amount of time dedicated to language learning. Additionally, the impact of EI on language achievement was consistent across various L1s, supporting the notion that EI affects language achievement in universal contexts (Wilce, 2009). However, the relationship was stronger when English was the target language compared to Spanish, possibly due to higher emotional comprehension in English-speaking learners (Downs et al., 2007).

Different language *achievement skills* also influenced the magnitude of the relationship. Stronger relationships were found in receptive skills (e.g., listening and reading) compared to general skills, speaking, vocabulary, and writing. This may be due to the higher likelihood of anxiety affecting receptive skills, thereby increasing the role of EI in these areas (Zhang, 2023; Ates, 2019). The analysis showed no significant differences in the EI-language achievement relationship across different *achievement measures*, suggesting that credits and GPA are also stable indicators of achievement. Finally, with regard to publication year, more recent studies indicated a stronger relationship between EI and language achievement. This trend may be attributed to the increased pressure associated with high-stakes testing, which amplifies the necessity for EI in managing academic-related emotions (Nichols et al., 2006). These findings underscore the escalating significance of EI in the context of language learning.

The findings of this meta-analysis offer several pedagogical implications for language educators seeking to enhance learners’ EI and academic success. First, a structured, step-by-step model such as the “ARGUER” framework—Awareness, Recognizing, Generating, Understanding, Expressing, and Regulating—can be incorporated into classroom activities to systematically foster learners’ EI (Li and Xu, 2019). For instance, teachers can integrate emotional reflection tasks, peer-based emotion recognition games, and classroom discussions that promote understanding and appropriate expression of emotions. Such practices align with previous research highlighting the critical role of classroom-based EI training in improving academic and emotional outcomes (Li and Xu, 2019). Second, the analysis indicates that EI plays a stronger role in earlier educational stages (e.g., secondary school, undergraduate level) than at the postgraduate level. Accordingly, language educators should adopt developmentally sensitive approaches to EI instruction, such as emotion-based storytelling or peer collaboration in younger learners, and metacognitive strategies to support emotion regulation in more advanced learners. This differentiation is consistent with educational psychology research that emphasizes the need for age-appropriate emotional skill development (Rivers et al., 2013). Third, given that EI exerts a more significant influence on receptive skills such as listening and reading, language instruction should embed emotion regulation techniques—such as stress-reduction strategies and positive self-talk—into these skill areas. This is particularly relevant in high-stakes or test-oriented environments, where learners often experience heightened anxiety (MacIntyre and Gregersen, 2012). Together, these suggestions provide actionable strategies for embedding EI into language pedagogy, thereby enhancing both emotional resilience and academic performance.

## 7 Limitations and direction for further research

This study provides robust evidence for understanding the relationship between EI and language achievement, reinforcing the notion that emotional factors are integral to language learning. While the findings are informative, several considerations should be acknowledged. First, some moderating variables—such as less-represented educational levels, learners with mixed majors, Spanish as the target language, and specific language skills—were underreported in the included studies. Similarly, incomplete demographic information such as gender distribution was observed in several sources. These gaps in the existing literature may slightly limit the interpretability of moderator analyses and reduce the precision of subgroup comparisons.

Second, although this meta-analysis extended beyond previous reviews by including both English- and Chinese-language publications, its linguistic scope remains potentially limited, as studies published in other languages were not incorporated. This may result in the omission of relevant findings from non-English and non-Chinese research contexts, which could potentially influence the comprehensiveness of the synthesized evidence. Third, this meta-analysis was based on cross-sectional data. While such data allow for the synthesis of a large and diverse sample, enhancing representativeness, they cannot capture the long-term influence of EI on language achievement. Despite these potential limitations, the present study offers several notable strengths. It systematically synthesizes data from a wide range of populations and learning contexts, incorporates both subjective and objective measures of achievement, and explores key moderator variables that enrich the understanding of how EI relates to language learning. These contributions provide a meaningful foundation for advancing the integration of emotional dimensions in language education research.

Future research on the relationship between EI and language achievement is encouraged to expand in several key areas. Firstly, future studies should consider broadening the geographical scope, as the current body of research is predominantly based in Asian countries (e.g., Iran, China, and Malaysia). Since EI may vary with environmental and cultural contexts (Schutte, 2014), it is crucial to validate these relationships in diverse regions, including Europe, the Americas, Africa, and other continents. Secondly, given that more than half of the studies focus on undergraduate students, future research should include participants from a broader range of educational levels to provide a more comprehensive understanding of the role of EI across different learning stages.

Thirdly, there is a need for greater attention to languages other than English as the target language. Although English learners comprise a significant portion of the global population, more research is necessary to determine whether the relationship between EI and language achievement holds in non-English language contexts. Fourthly, future studies should address the current imbalance in the focus on specific language skills. Examining the role of EI in the context of particular language skills could provide deeper insights into its functioning and effects. Fifthly, further exploration is needed into the broader role of EI in language learning, especially since the results suggest that the impact of EI on language achievement increases over time.

Finally, future research should adopt more refined and diverse analytical approaches. While correlation analysis remains common due to its simplicity (Norouzian and Plonsky, 2018), advanced methods such as regression analysis and structural equation modeling offer greater potential for exploring complex relationships and predictive modeling (Plonsky and Oswald, 2017). Given the multifaceted nature of language achievement, incorporating learner-internal variables through person-centered approaches—such as latent profile analysis—may yield deeper insights into how EI functions across different learner subgroups. Moreover, longitudinal designs are equally important, as they enable researchers to examine the sustained impact of emotional intelligence on language achievement over time.

## 8 Conclusion

This meta-analysis provides a comprehensive examination of the association between EI and language achievement, underscoring the significance of emotional factors in language learning. The findings reveal a positive, albeit small, relationship between EI and subjective achievement, and a more pronounced, moderate relationship with objective achievement. Moreover, the positive impact of EI on objective achievement was found to vary according to *educational level, target language, achievement skill*, and *publication year*. These nuanced insights emphasize the role of EI in enhancing language learning outcomes, thereby contributing to a more holistic approach to foreign language education that values emotional as well as cognitive development.

By highlighting these dynamics, the study offers valuable directions for future research aimed at promoting the sustainable development of foreign language education. By expanding our understanding of how EI influences language achievement across different contexts and learner variables, this study provides a foundation for developing more effective and inclusive language education practices that address both emotional and cognitive dimensions, fostering a more balanced and sustainable educational environment.

## Data Availability

The data analyzed in this study is subject to the following licenses/restrictions: the data can be accessed by contacting the corresponding author. Requests to access these datasets should be directed to lishuhong98@163.com.
